# Comprehensive analysis of housekeeping genes, tissue-specific genes, and dynamic regulation across developmental stages in pearl millet

**DOI:** 10.1186/s12864-024-11114-3

**Published:** 2024-12-18

**Authors:** Wei Luo, Min Sun, Ailing Zhang, Chuang Lin, Yarong Jin, Xiaoshan Wang, Linkai Huang

**Affiliations:** 1 College of Grassland Science and Technology, Sichuan Agricultural University, Chengdu, 611130 China; 2 Institute of Advanced Study, Chengdu University, Chengdu, 610106 China

**Keywords:** Pearl millet, Housekeeping gene, Tissue-specific gene, RNA-seq, Development

## Abstract

**Background:**

Pearl millet (*Pennisetum glaucum* (L.) R. Br.) is a vital cereal crop, predominantly cultivated in arid and semi-arid regions of Asia and Africa. It serves as a staple food for millions, while also being utilized as forage and an energy crop. The crop’s resistance to heat and drought, coupled with its high biomass, positions it as a promising candidate for climate-resilient agriculture. A detailed understanding of its gene expression patterns across various tissues and developmental stages is essential for enhancing its yield and quality. This study aims to fill this knowledge gap by employing RNA-seq to identify housekeeping genes (HKGs) and tissue-specific genes (TSGs) in pearl millet.

**Results:**

Our analysis of RNA-seq data from nine tissues (seed, germ, radicle, leaf, root, tillering tissue, stem, spike, and grain) across eight developmental stages in pearl millet accession Tifleaf3 revealed a comprehensive gene expression profile. We identified 461 HKGs that exhibited stable expression across all tissues and stages, providing robust internal references for RT-qPCR. Additionally, 8091 TSGs were discovered, many of which showed distinctive expression patterns in tissues such as spike, stem, and leaf. Functional enrichment analysis of these genes using GO and KEGG pathways highlighted their roles in key biological processes and pathways, indicating their potential in crop trait enhancement. Protein-protein interaction networks constructed for stem and leaf tissues further illuminated the regulatory mechanisms underlying the transition from vegetative to reproductive growth stages.

**Conclusion:**

This study presents a detailed transcriptomic landscape of pearl millet, identifying a set of HKGs and TSGs that are crucial for understanding the molecular basis of its growth and development. We provided valuable options for transcript normalization and crucial targets for exploring gene function for the plant growth and development in pearl millet. The insights gained from this work are instrumental for breeding programs aimed at enhancing the productivity of pearl millet, thereby contributing to food and energy security.

**Supplementary Information:**

The online version contains supplementary material available at 10.1186/s12864-024-11114-3.

## Background

Pearl millet (*Pennisetum glaucum* (L.) R. Br.) is a versatile plant used for various purposes, including as food, forage, and energy crop. It ranking as the sixth most important cereal crop globally after rice, wheat, maize, barley, and sorghum, serves as a staple food source for 90 million people in the arid and semi-arid tropical regions of Asia and Africa [1]. It is also being recognized as a warm-season annual forage grass in many countries for its high biomass and nutritive quality [2, 3]. Furthermore, high biomass is considered as an advantage for energy crop, and pearl millet grain is an emerging source of starch for industrial applications [4, 5]. Pearl millet exhibits strong resistance to heat and drought stress, presenting promising potential in ensuring food and forage, as well as energy security amidst climate change challenges [6, 7]. Recognizing the crop’s significance, its genome, pan-genome, and multi-omics database have been published, offering extensive resources for enhancing pearl millet [8–11]. The identification of genes associated with key traits in pearl millet will play a crucial role in enhancing its yield and quality in the future [12]. For example, *d2* is found responsible for plant height [13], and *bmr* is reported to reduce lignin content in pearl millet [14].

Real-time quantitative polymerase chain reaction (RT-qPCR) is a crucial experiment in evaluating gene function, known for its accurate assessment of target gene expression. To ensure data reliability, a normalization strategy is employed, commonly comparing expression levels of the target gene with an internal reference gene in the same sample [15]. So-called housekeeping genes (HKGs) are universally used as internal reference for normalization in RT-qPCR expression analysis [16]. For example, *actin* (*ACT*), *glyceraldehyde-3-phosphate dehydrogenase* (*GAPDH*), *tubulin* (*TUB*), *ubiquitin-conjugating enzyme* (*UBC*), *polyubiquitin* (*UBQ*) and *cyclophilin* (*CYP*) are commonly used HKGs for traditional internal reference genes (TIRGs), for their stable expression and essential cellular roles [17, 18]. However, some TRIGs have been found to be regulated under certain conditions, indicating that they may not be stably expressed in model plant *Arabidopsis thaliana* [19]. This highlights the importance of identifying the most suitable internal reference genes for specific plant species in order to ensure accurate RT-qPCR expression analysis.

HKGs, as defined by She et al. [20], are genes that are constitutionally expressed in all tissues with relatively consistent expression levels across various cell types. In contrast, tissue-specific genes (TSGs) are predominantly expressed in a single type of tissue and show low or no expression in others. Typically, TSGs closely align with the specific functions of their respective tissue types [21]. By analyzing the expression patterns of TSGs, researchers can gain insights into the regulatory networks that govern plant growth and development, as well as the roles of genes in various biological processes [22, 23]. The sequence data of TSGs are essential for designing tissue-specific promoters, which are DNA sequences that regulate gene expression in specific tissues or cell types and play a crucial role in developing genetically modified crops with enhanced traits [24–26]. Understanding TSGs offers valuable information on the genetic foundations of complex traits and assists in devising strategies to enhance crop productivity.

RNA-seq is a sensitive and reliable tool for identifying genes with specific expression patterns [27]. It is an ideal strategy not only for identifying HKGs suitable for use as internal reference genes in RT-qPCR, but also widely used to identify TSGs in various plant and animal species. Through the analysis of RNA-seq data from multiple tissues, a significant number of HKGs and TSGs have been identified in species such as *A. thaliana* [28], *Cicer arietinum* [29], *Chenopodium quinoa* [30], *Zea mays* [31] and *Glycine max* [32]. The identification of HKGs and TSGs holds promise for advancing research on gene function in pearl millet.

In this study, RNA-seq data was analyzed from 9 tissues (seed, germ, radicle, leaf, root, tillering tissue, stem, spike and grain) across 8 developmental stages (imbibition, three-leaf, five-leaf, tillering, flowering, heading, dough, and ripening) in pearl millet to investigate the relationship and unique characteristics of gene expression patterns among different tissues at various developmental stages. The findings offer comprehensive insights into the growth and development processes of different tissues, particularly those with significance in food, forage and energy, such as spike, stem, and leaf. The identification of HKGs in this research presents more reliable options for normalization in RT-qPCR. Moreover, the study highlights the significance of TSGs, as well as genes that are consistently and differentially expressed in spike, stem, leaf, and root, as crucial targets for analyzing gene function in pearl millet.

## Methods

### Plant materials and RNA-seq data

Transcriptome analysis was conducted on multiple tissues of pearl millet accession Tifleaf3 at various growth stages. Seeds of Tifleaf3 were placed on a double-layer filter paper in a petri dish for germination, allowing for sampling of imbibed seed, germ, and radicle [33]. Seedlings were then grown in a greenhouse under normal conditions at the College of Grassland Science and Technology, Sichuan Agricultural University, Chengdu, China. Samples were collected at different stages of development, including three-leaf and five-leaf stages for leaf and root, tillering stage for leaf, stem, tiller tissue, and root, heading, flowering, and dough stages for spike, leaf, and stem, and ripening stage for grain [10]. A total of 24 tissues with three biological replicates from differently developmental stages (TDDSs, Table 1) were collected to extract RNA with RNeasy Plant Mini Kit according to the manufacturer’s instructions. RNA concentration of each sample was determined using NanoDrop Onec (Thermo Fisher Scientific, USA), and RNA integrity was verified by electrophoresis in 1% agarose gel.

**Table 1:** Sampled tissues and the stages

Stages	Tissues	Abbreviations for sampled tissues
Imbibition	Seed	I0
Imbibition after 24 h	Seed	I24
Imbibition after 36 h	Germ	I36G
Imbibition after 36 h	Radicle	I36R
Imbibition after 48 h	Germ	I48G
Imbibition after 48 h	Radicle	I48R
Three-leaf stage	Leaf	3LL
Three-leaf stage	Root	3LR
Five-leaf stage	Leaf	5LL
Five-leaf stage	Root	5LR
Tillering stage	Leaf	TL
Tillering stage	Young tiller	TT
Tillering stage	Stem	TST
Tillering stage	Root	TR
Heading stage	Spike	HSP
Heading stage	Leaf	HL
Heading stage	Stem	HST
Flowering stage	Spike	FSP
Flowering stage	Leaf	FL
Flowering stage	Stem	FST
Dough stage	Spike	DSP
Dough stage	Leaf	DL
Dough stage	Stem	DST
Ripening stage	Grain	RG

The construction of the libraries was facilitated by the NEBNext UltraTM RNA Library Prep Kit for Illumina. Initially, the mRNA was enriched using the NEBNext Poly (A) mRNA Magnetic Isolation Module. Subsequently, fragment buffer was introduced to break down the mRNA into shorter segments, followed by the synthesis of first strand cDNA using random hexamer primers. In the subsequent step, dNTPs, DNA polymerase I, and buffer were incorporated to generate the second strand cDNA. The double-stranded cDNA was then purified with AMPure XP beads, with end repair and sequencing adapter ligation performed. The size selection of the cDNA fragments was also carried out using AMPure XP beads. The cDNA library was subsequently amplified via PCR. Quality assessment and quantification of the cDNA library were conducted using the HT DNA High Sensitivity Assay Kit on the Caliper LabChip GX. RNA sequencing was executed on the Illumina HiSeq2000 platform.

Raw RNA-seq datasets can be accessed at the National Center for Biotechnology Information under project IDs PRJNA670183 and PRJNA850467. Clean RNA-seq data were obtained after the raw data filtered out the adapters and low-quality nucleotides sequences using Trimmmatic (Version 0.36) [34]. The quality of clean data was detected by FastQC [35], then the transcript per million (TPM) for each genes were calculated by Kallisto [36].

In addition, three biological replicates for 12 TDDSs, including the spike (heading, flowering, and dough stages), leaf (five-leaf, tillering, heading, flowering, and dough stages) and stem (tillering, heading, flowering, and dough stages), of accession Tift23A were collected to extract RNA and perform RT-qPCR.

### Systematic identification of HKGs and TSGs with RNA-seq data

In this research, gene expression was defined as having TPM values above 0.1 in all three replicates; otherwise, the gene was considered unexpressed and its TPM values were set to 0. The HKG criteria, based on the work of Machado et al. [32] and She et al. [20] with some adjustments, required genes to be expressed in all 24 TDDSs, have TPM values higher than the median of expressed genes in each TDDS, and have a coefficient of variation (CV) lower than the lower quartile value of expressed genes in 2–24 TDDSs.

Tissue specificity analysis for the 24 tissues at different developmental stages was conducted using the tissue specificity index (TAU) values ranging from 0 to 1 in TBtools software [37]. Genes with TAU values below 0.85 or equal to or greater than 0.85 were categorized as having widespread or tissue-specific expression, respectively [38]. A more stringent threshold of TAU greater than 0.9 was applied to identify tissue-specific expression genes in this study.

### Gene expression trend and differential expression analysis

Gene expression trend analysis in several important tissues, such as root, stem, leaf and spike, were performed by Mfuzz [39] on bioinformatics analysis platform BioLadder (https://www.bioladder.cn/). Differentially expressed genes (DEGs) in a tissue across different growth stages were analyzed using the Limma package [40] on bioinformatics analysis platform Sangerbox [41]. DEGs were identified based on the criteria of a log_2_^fold − change^ < − 2 or > 2 and a *p*-value < 0.05.

### RT-qPCR validation

cDNA of Tift23A was synthesized using a HiScript III All-in-one RT SuperMix Perfect for qPCR (Vazyme, China) according to the instructions and was stored at -20 ℃. Three biological replicates for each sample of accession Tift23A were used for RT-qPCR analysis with three technical replicates. RT-qPCR was performed using a QX96M Real-time PCR System (JLM, China). The reactions were carried out using Taq SYBR Green-based qPCR Premix (Universal) following the manufacturer’s instructions.

Expression stability of the 7 HKGs and 4 HKG-TIRGs across different growth stages and tissues in accession Tift23A were determined using RefFinder [42] as well as others four statistical algorithms, namely geNorm [43], Normfinder [44], BestKeeper [45], and the comparative Δ-Ct method [46].

The expression patterns of 10 DEGs involved in the development of leaf tissues were analyzed using RT-qPCR across leaf samples from the five-leaf, tillering, heading, flowering, and dough stages. The relative expression was assessed using the 2^−ΔΔCt^ method, with the most stable gene among the 11 (7 HKGs and 4 HKG-TIRGs) serving as the internal reference.

### Functional enrichment and protein-protein interaction analysis

Functional analysis of HKGs, TSGs, and DEGs included Kyoto Encyclopedia of Genes and Genomes (KEGG) and Gene Ontology (GO) enrichment, which was carried out using the KEGG/GO Enrichment module on the Milletdb platform, utilizing gene annotation data from the pearl millet accession PI537069 [10]. Additionally, a list of pearl millet transcription factors (TFs) for TSGs was predicted by iTAK [47]. Moreover, the protein-protein interaction (PPI) analysis was conducted based on the STRING database [48] with default parameters and reference of *Oryza sativa Indica*.

## Results

### Gene expression profile based on illumina sequencing

**Fig. 1:**
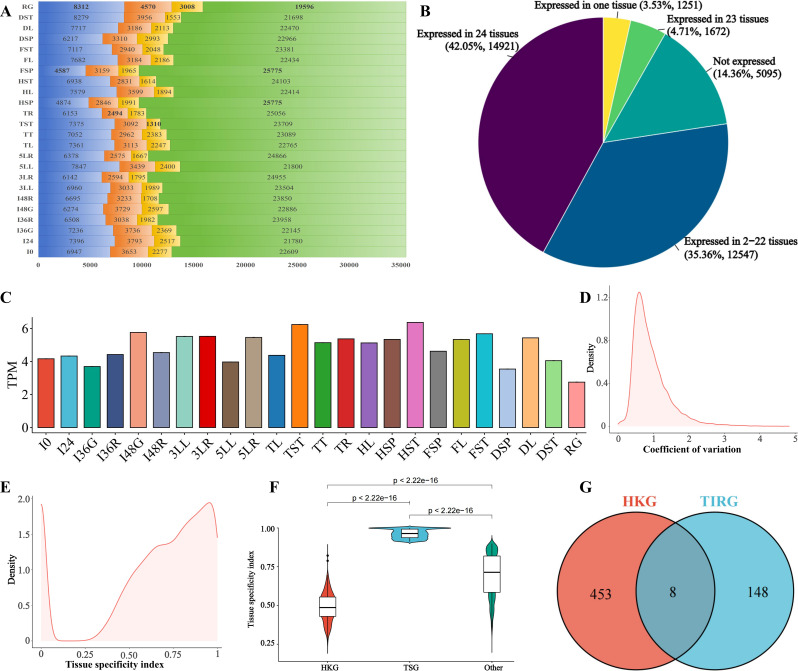
Expression of the 35,486 genes in the 24 TDDSs, and identification of HKGs and TSGs. **A**, Genes were categorized into four groups based on their expression intensity and stability across biological replicates: genes with three replicates of TPM equal to 0 (blue), genes with 1–2 replicates of TPM equal to 0 (orange), genes with three replicates of TPM values greater than 0 and less than or equal to 0.1 (yellow), and genes with three replicates of TPM values greater than 0.1 (green). The numbers indicate the gene count, with bold numbers highlighting the maximum or minimum values in each group. **B**, The number and percentage of expressed genes across the 24 TDDSs. **C**, The median of TPM values for each TDDSs. **D**, Density of CV for 29,140 genes which expressed in 2–24 TDDSs. **E**, Density of tissue specificity index for 35,486 genes. **F**, Violin plot showing the distribution of TAU scores of housekeeping, tissue-specific and the remaining genes. **G**, Venn diagram for the HKGs and TIRGs

In this study, raw reads ranging from 34,229,066 to 52,243,135 were obtained, and clean reads filtered from 33,355,566 to 51,180,780 were analyzed for the 72 sequenced samples (Table S1). A total of 417.76 Gb clean bases were utilized for downstream analysis (Table S1). In the genome of pearl millet accession PI537069, 35,486 genes were annotated, exhibiting varying expression levels across different TDDSs. Within the 24 TDDSs, TPM values in the three biological replicates ranged from 4587 (FSP) to 8312 (RG) genes with a value of 0, while TPM values in one or two biological replicates ranged from 2494 (TR) to 4570 (RG) genes with a value of 0. The number of genes with 0 < TPM ≤ 0.1 in the three biological replicates varied from 1310 (TST) to 3008 (RG) genes, with these genes considered unexpressed and their TPM values set to zero (Fig. 1A, Table S2). Genes with TPM values greater than 0.1 in all three biological replicates, totaling 19,596 (RG) to 25,775 (FSP and HSP) genes, were classified as expressed genes (Fig. 1A, Table S2). This resulted in 42.05% (14921 genes) being expressed in all 24 TDDSs, 4.71% (1672 genes) in 23 TDDSs, 3.53% (1251 genes) in only one TDDS, 14.36% (5095 genes) not being expressed in any tissue, and the remaining genes being expressed in 2–22 tissues (Fig. 1B, Table S2).

### 461 genes meet the criteria for HKGs in pearl millet

HKGs in pearl millet were identified by filtering for both constitutively and robustly expressed genes. Out of the 14,921 genes expressed in 24 TDDSs, 1427 genes had TPM values higher than the median (ranging from 2.75 to 6.40, Fig. 1C). The CV for the 29,140 genes expressed in 2–24 TDDSs ranged from 0.0002 to 4.83 (Fig. 1D), with 461 genes showing a CV lower than 0.57 (lower quartile value). This indicates that 461 HKGs were identified in pearl millet (Table S3), showcasing low expression variation and consistent expression levels. TAU scores, ranging from 0 to 1 (Fig. 1E), revealed that the HKGs had TAU values between 0.26 and 0.82 (Fig. 1F, Table S3), supporting their stable and generalized expression levels.

Among the 461 HKGs identified, 15 were annotated across 10 families of TFs, including *AP2* (*PMA2G07260.1*), *B3* (*PMA2G04990.1*), *bHLH* (*PMA4G00242.1* and *PMA7G01261.1*), *C2H2* (*PMA7G01029.1*), *C3H* (*PMA2G02384.1*), *GRF* (*PMA7G01755.1*), *HB* (*PMA2G06053.1* and *PMA7G04036.1*), MADS (*PMA6G06744.1*), *NF-Y* (*PMA1G02893.1*, *PMA7G04772.1*, *PMA1G01749.1* and *PMA4G04255.1*) and *Whirly* (*PMA2G00345.1*). These transcription factors have all been reported to be implicated in plant growth and development, possessing prominent characteristics of HKGs. For instance, *GRF* is a plant-specific transcription factor known for its involvement in root, stem, and leaf development, as well as flower and seed formation [49].

**Fig. 2:**
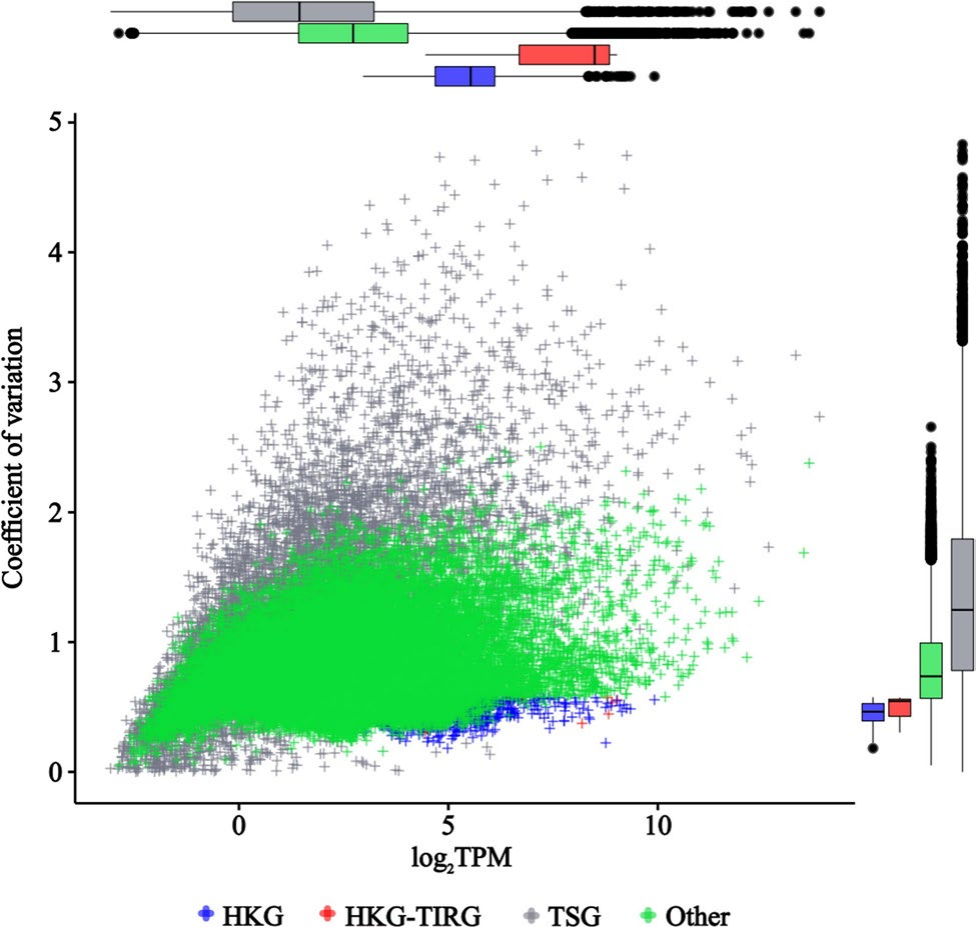
Scatter plot of CV-log_2_TPM for the 29,140 genes expressed in 2–24 TDDSs

**Fig. 3:**
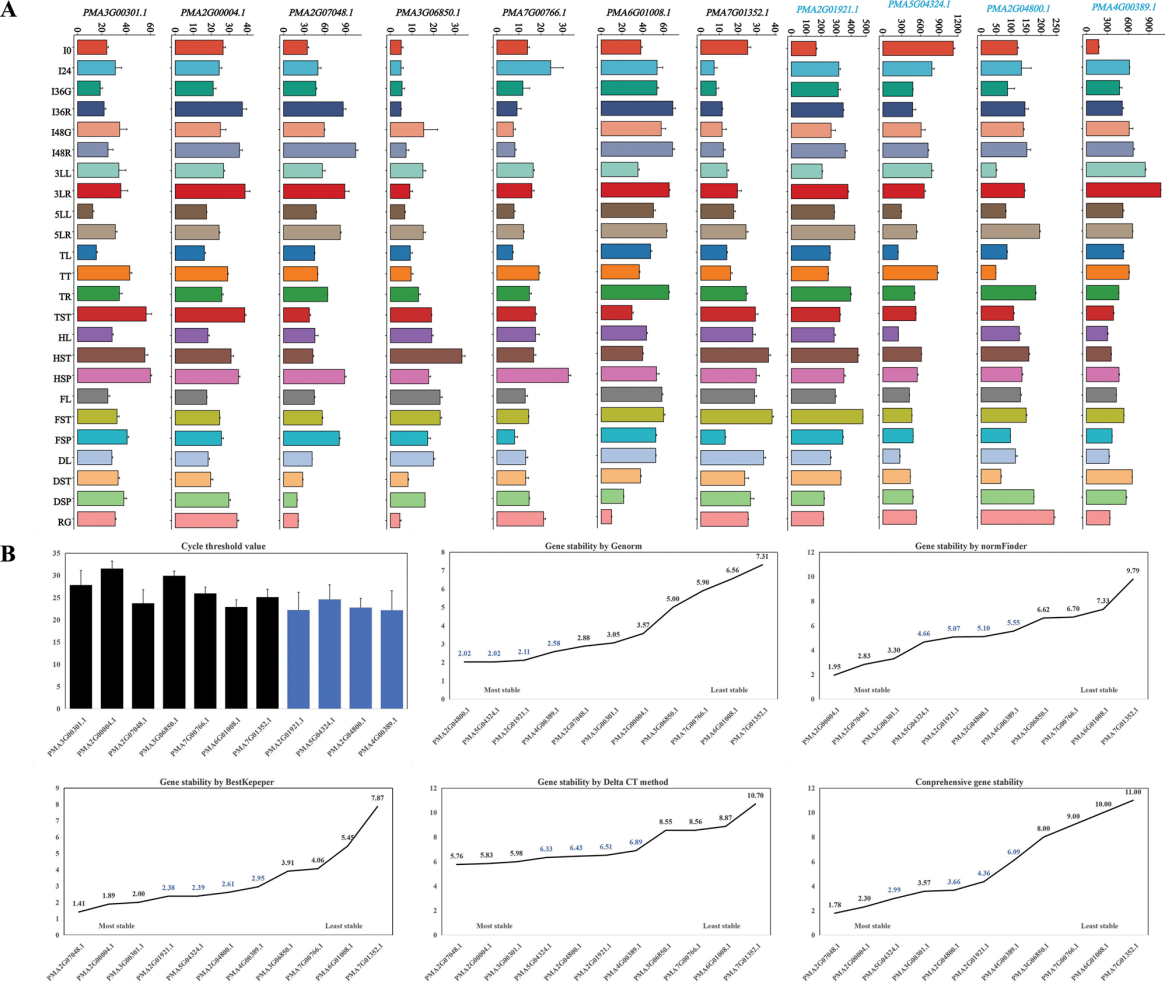
Expression patterns and stability of HKGs and HKG-TIRGs. **A**, Expression patterns of 7 HKGs (black ID) and 4 HKG-TIRGs (blue ID) in 24 TDDSs. **B**, Comparison of the expression stability for the 7 HKGs (black bar or number) and 4 HKG-TIRGs (blue bar or number) with cycle threshold value using Delta CT method, BestKeeper, NormFinder, Genorm and RefFinder

In pearl millet, 18 *ACT*, 30 *CYP*, 9 *GAPDH*, 23 *TUB*, 74 *UBC*, and 2 *UBQ* genes were annotated (Table S4), with 8 of them, including 6 *UBCs* (*PMA2G01921.1*, *PMA2G04800.1*, *PMA7G06369.1*, *PMA1G05188.1*, *PMA6G00452.1* and *PMA4G00600.1*), 1 *GAPDH* (*PMA4G00389.1*) and 1 *UBQ* (*PMA5G04324.1*), being identified as HKGs in this study and labeled as HKG-TIRGs (Fig. 1G). These HKG-TIRGs did not exhibit the most intense and stable expression levels compared to other HKGs (Figs. 2 and 3).

The stability of 11 randomly selected genes (Fig. 3A), comprising 7 HKGs and 4 HKG-TIRGs (primers listed in Table S5), was assessed using various statistical methods based on the cycle threshold values from the RT-qPCR in 12 TDDSs of Tift23A. The mean cycle threshold values of 11 potential reference genes ranged from 22.17 (*PMA4G00389.1*) to 31.53 (*PMA2G00004.1*) (Fig. 3B). The 4 HKG-TIRGs were identified as the most stable genes by Genorm (Fig. 3B). However, *PMA2G07048.1*, *PMA2G00004.1*, and *PMA3G00301.1* were selected as the most stably expressed genes by three algorithms (Fig. 3B), namely Normfinder, BestKeeper, and the comparative Δ-Ct method. The comprehensive analysis from RefFinder indicated that *PMA2G07048.1* was the most stable gene.

**Fig. 4:**
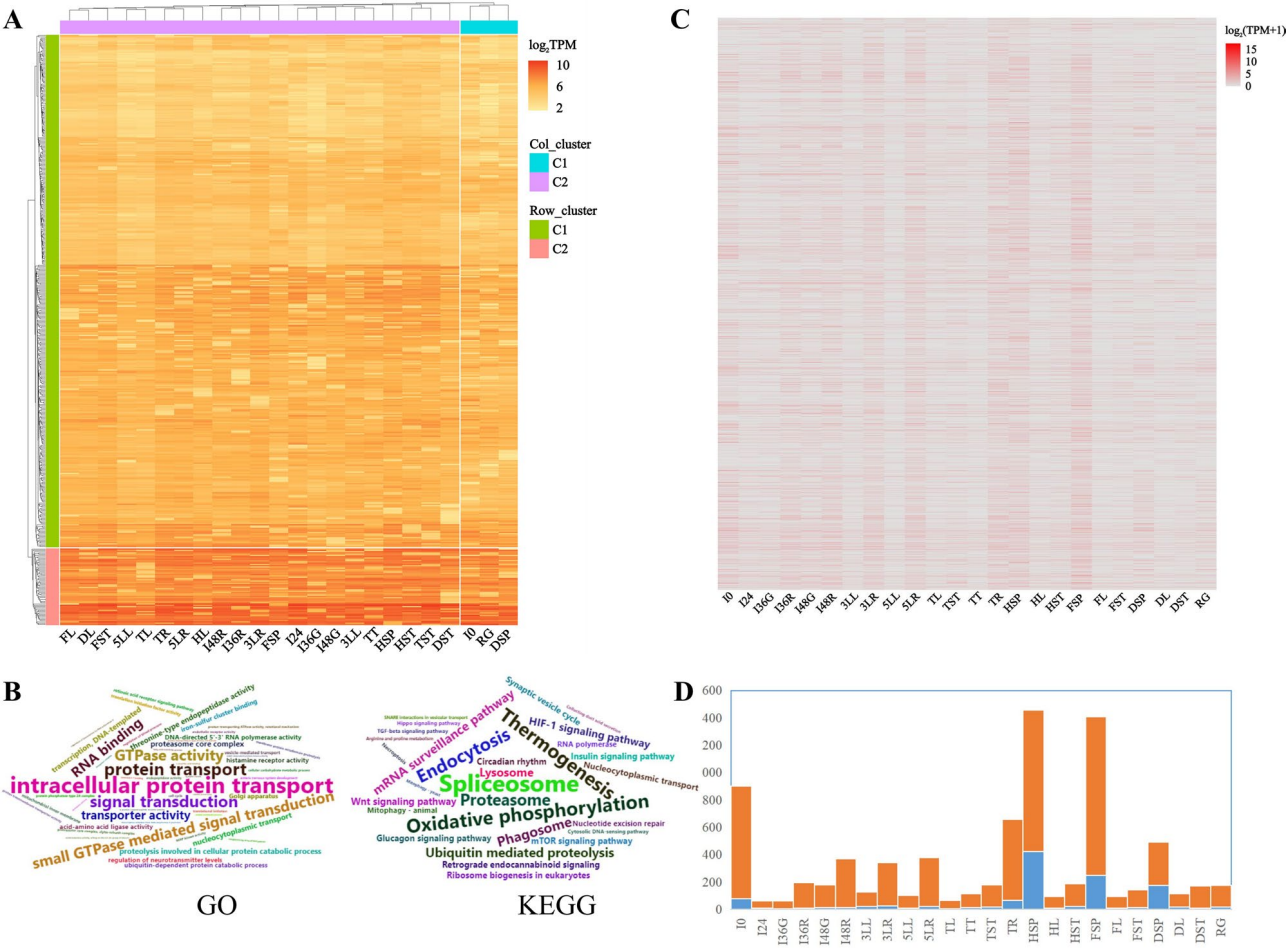
Global expression patterns of HKGs and TSGs. **A**, Global expression patterns of HKGs. The clusters were grouped based on gene expression levels for HKGs. **B**, Word cloud for significantly enriched GO terms and KEGG pathways for HKGs. The font size of the word cloud reflects the number of enriched genes linked to each term (pathway), with larger fonts indicating a greater gene count and smaller fonts indicating fewer genes. Only terms (pathways) with a *p*-value < 0.05 are exhibited. **C**, Global expression patterns of TSGs. **D**, Column chart of TSGs in 24 TDDSs. Blue and orange indicate the gene number with 0.9 < TAU < 1 and TAU = 1, respectively

The expression levels of HKGs were categorized into two main clusters, with three *UBC* genes falling into Row_cluster 1 and other five TIRGs into Row_cluster 2 (Fig. 4A). Similarly, the 24 TDDSs were also divided into two clusters, with 21 and 3 TDDSs (I0, RG, and DSP) in the two Col_cluster 1 and 2, respectively (Fig. 4A).

GO enrichment analysis of the 461 HKGs revealed a diverse range of functions, with 51 significantly enriched terms (*p*-value < 0.05, Fig. 4B), including intracellular protein transport, protein transport, signal transduction, small GTPase mediated signal transduction, GTPase activity, RNA binding and transporter activity. 30 KEGG pathway were significantly enriched (*p*-value < 0.05, Fig. 4B), such as spliceosome, thermogenesis, oxidative phosphorylation, endocytosis, proteasome, phagosome, ubiquitin mediated proteolysis, mRNA surveillance pathway, lysosome, protein processing in endoplasmic reticulum and HIF-1 signaling pathway.

### Significant specific developmental features were shown in 8091 TSGs

A total of 8091 genes with TAU > 0.9 exhibited a distinct tissue-specific pattern, with many of these genes showing high expression in one TDDS while being low or not expressed in others (Figs. 1F, 2 and 4C, Table S6). Furthermore, a scatter plot of CV-log_2_TPM revealed the presence of distinct types of tissue-specific genes, as illustrated by the gray plots in Fig. 2: some with low CV and expression level, and others with high CV and intense expression.

Out of the 8091 TSGs, 1251 genes had a TAU value of 1.0, indicating exclusive expression in a single TDDS. Among the TDDSs, I36G and HSP had the lowest (5) and highest (422) number of TSGs with TAU = 1.0, respectively (Fig. 4D, Table S6). Similarly, I24 and FSP had the least (54) and most (1160) TSGs with TAU values between 0.9 and 1.0, respectively (Fig. 4D, Table S6). Notably, spike tissues such as HSP and FSP exhibited the highest number of TSGs (Fig. 4D, Table S6). The leaf tissues (3LL, 5LL, TL, HL, HL, and DL), spike tissues (HSP, FSP, and DSP), stem tissues (TST, HST, FST, and DST), root tissues (3LR, 5LR, and TR), and radicle tissues (36R and 48R) exhibited notable variations in the abundance of TSGs. Interestingly, the number of TSGs did not directly correlate with the number of TDDS for each organ, underscoring distinct patterns of TSG expression across different parts of the plant (Fig. 4D, Table S6).

A total of 572 TSGs, accounting for 7.08% of the total, were characterized across 36 families of TFs as detailed in Table S7. Among all investigated samples, I0 exhibited the highest diversity with 24 distinct TF types and the largest number of TFs (102), while only a bHLH TF was identified in I24 (Table S7). Notably, spike tissues demonstrated the highest abundance of tissue-specific TFs, surpassing leaf, root, and stem tissues in the dataset (Table S7). The AP2 and bHLH TF families emerged as the top two families with the most tissue-specific TFs, showing broad representation across diverse TDDSs (Table S7). The TSGs belonging to the bZIP, GRAS, HB, WRKY, MADS, C2H2, NAC, and MYB gene families were found in 10 or more TDDSs, indicating their widespread involvement in developmental processes across pearl millet samples (Table S7). The gene expression profiles within these families encompass the entire developmental span of pearl millet, covering both vegetative and reproductive growth stages.

**Fig. 5:**
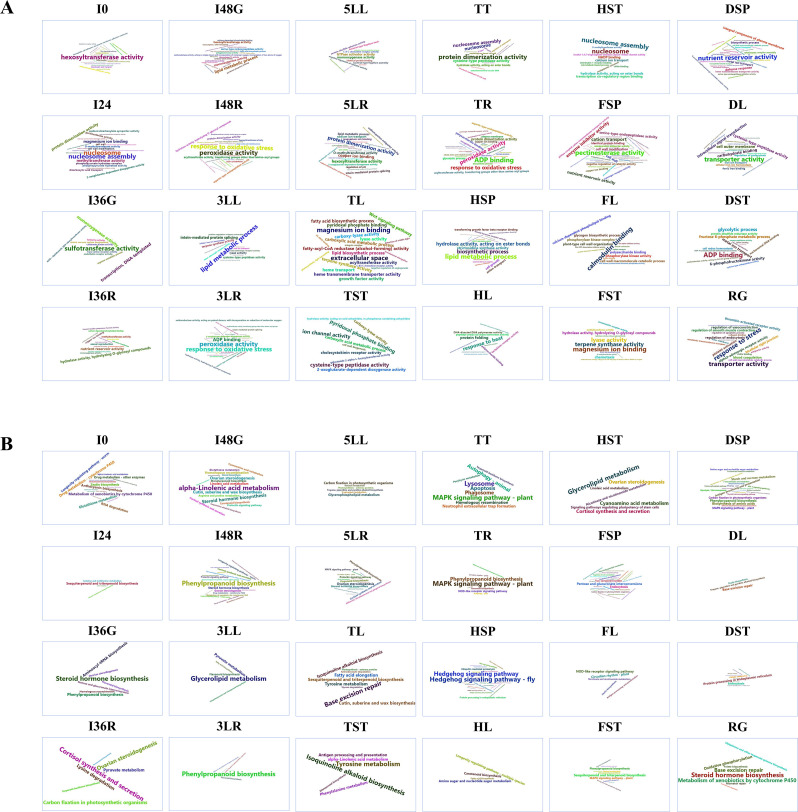
Word clouds depicting significantly enriched GO terms (**A**) and KEGG pathways (**B**) for TSGs. The font size of the word cloud corresponds the number of enriched genes linked to each term (pathway), with larger fonts signifying a higher gene count and smaller fonts indicating fewer genes. Only terms (pathways) with a *p*-value < 0.05 are displayed

The results of GO enrichment analysis for TSG revealed differences in enriched terms across different developmental stages, even within the same organ (Fig. 5A). For instance, six and four growth stages of leaf and stem development do not significantly enrich the same terms (Fig. 5A), respectively. However, three stages of root development shared two common enriched terms: acyltransferase activity (transferring groups other than amino-acyl groups) and oxidoreductase activity (acting on paired donors, with incorporation or reduction of molecular oxygen). Interestingly, while the three spike development stages did not share significantly enriched terms, TSGs from FSP and DSP both enriched five terms, including enzyme inhibitor activity, pectinesterase activity, sexual reproduction, nutrient reservoir activity, and phosphorelay response regulator activity (Fig. 5A). Notably, terms such as sexual reproduction and nutrient reservoir activity exhibited significant developmental characteristics, potentially linked to grain formation and development (Fig. 5A).

No KEGG pathway was found to be enriched for the TSGs of pearl millet in most tissues across different growth stages, including leaf, stem, and spike (Fig. 5B). However, three KEGG pathways (flavonoid biosynthesis, phenylpropanoid biosynthesis, stilbenoid, diarylheptanoid, and gingerol biosynthesis) were significantly enriched in the TSGs from the three growth stages of the root (Fig. 5B). Additionally, TSGs from FSP and DSP both showed significant enrichment in two KEGG pathways, carbon fixation in photosynthetic organisms, and pentose phosphate pathway, while TSGs from HSP did not show enrichment in these pathways (Fig. 5B).

### Construction of PPI networks for development of stem and leaf

Stem and leaf are pivotal tissues in pearl millet, with gene expression patterns in these tissues providing valuable insights into pearl millet development. An analysis of stem tissue across different stages revealed 20,056 co-expressed genes organized into 12 clusters, with clusters 4 and 8 having the highest gene counts and cluster 11 the lowest (Fig. S1). Genes in cluster 6 exhibited a gradual increase in expression levels. Clusters 2, 5, and 12 showed a sustained decrease in expression levels from tillering to maturity, with differing degrees of decline. Clusters 2 and 5 demonstrated a slow decrease, while cluster 12 displayed a linear decrease. Genes in clusters 1 and 10 maintained stable expression levels during the reproductive growth stage, but genes in cluster 1 decreased from TST to this stage (HST, FST, and DST), while genes in cluster 10 increased (Fig. S1).

A total of 19,619 genes expressed across six leaf developmental stages including, three-leaf, five-leaf, tillering, heading, flowering, and dough stages, were categorized into 16 clusters based on expression patterns (Fig. S2). Genes were evenly distributed among clusters, with the highest and lowest gene counts being 1418 (cluster 9) and 887 (cluster 4), respectively. Leaf clusters did not exhibit sustained up- or down-regulation, but genes in clusters 4 and 7 maintained stable expression levels during both vegetative and reproductive growth stages. Cluster 4 showed higher expression levels during vegetative growth, while cluster 7 exhibited the opposite pattern (Fig. S2).

**Fig. 6:**
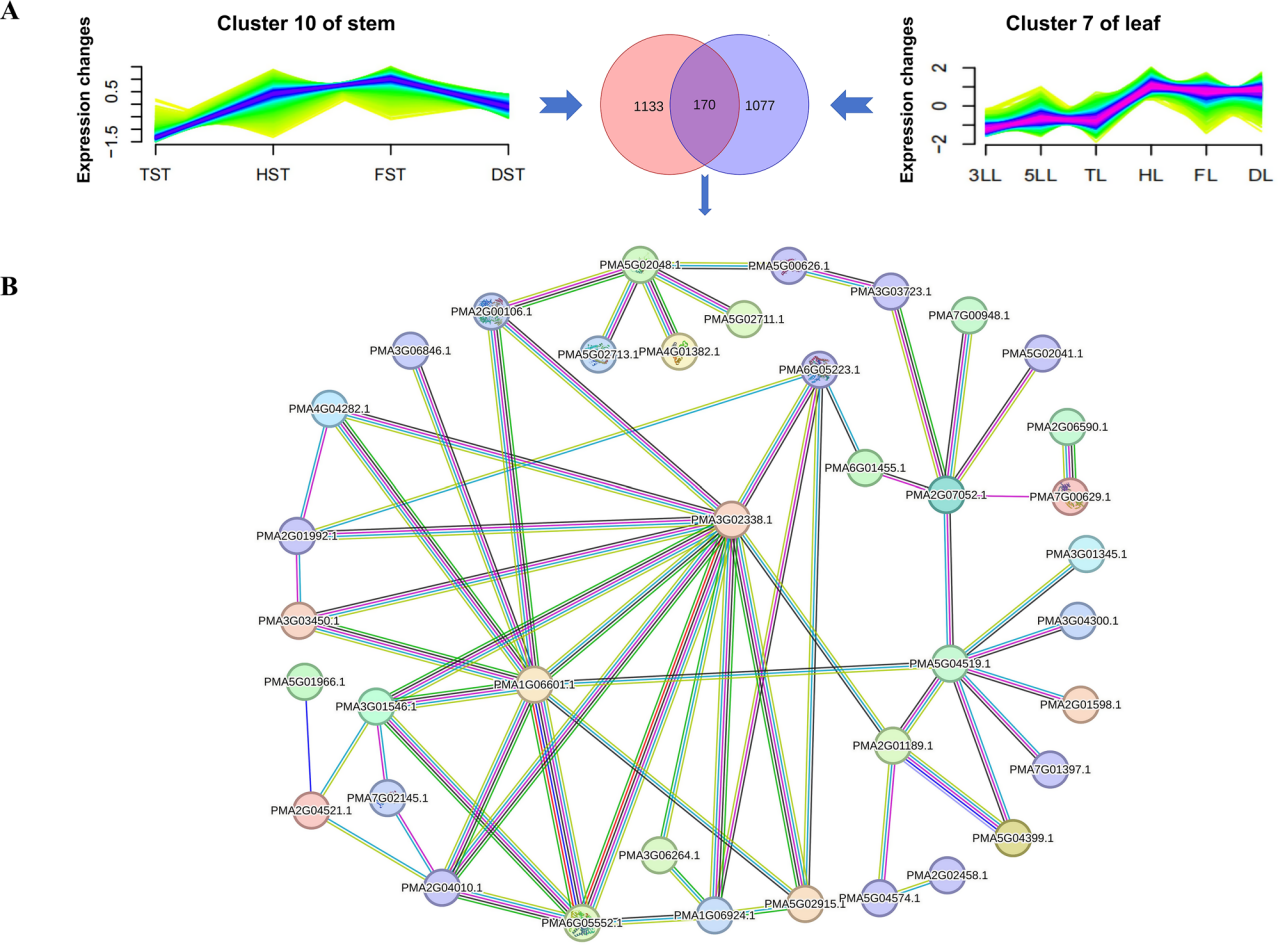
A protein-protein interaction network was constructed to investigate the development of stems and leaves during the transition from the vegetative to the reproductive growth stage. **A**, 170 genes were identified as up-regulated during the shift from the vegetative (tillering stage) to the reproductive (flowering stage) growth phases. **B**, A predicted interaction network encompassing 53 proteins potentially implicated in the development of both stem and leaf tissues. Line color indicates the type of interaction evidence. Bright blue, known interaction (curated database); pink, known interaction (experimentally determined); green, predicted interaction (gene neighborhood); red, predicted interaction (gene fusion); dark blue, predicted interaction (gene co-occurrence); green, potential interaction (text mining); black, potential interaction (co-expression); light blue, potential interaction (protein homology)

A total of 170 genes were clustered simultaneously between cluster 10 of stem and cluster 7 of leaf (Fig. 6A). Among these genes, 38 were identified to form a protein interaction network that may play a role in the signaling cascade governing the transition from nutrient to reproductive growth in pearl millet. The expression levels of these genes remained relatively stable across the two growth stages, exhibiting an increase during the transitional phase between the developmental stages. Core proteins encoded by *PMA3G02338.1*, *PMA1G06601.1*, *PMA6G05223.1*, *PMA5G04519.1* and *PMA2G07052.1* were found to be involved in pearl millet development within this PPI network (Fig. 6B).

Furthermore, gene expression trends in both spike and root tissues were analyzed. Analysis of spike tissue at various stages identified 21,373 co-expressed genes, which were classified into 6 clusters according to their expression profiles (Fig. S3). Clusters 1–3 exhibited a higher number of genes in comparison to clusters 4–6 (Fig. S3). Conversely, in the root tissue, a total of 23,477 genes were identified as co-expressed across the three-leaf, five-leaf, and tillering stages, and were organized into 8 clusters (Fig. S4). Cluster 7 displayed the highest gene count, while cluster 3 had the lowest (Fig. S4).

### DEGs for development of different tissues show significant enrichment of the same GO terms and KEGG pathways

**Fig. 7:**
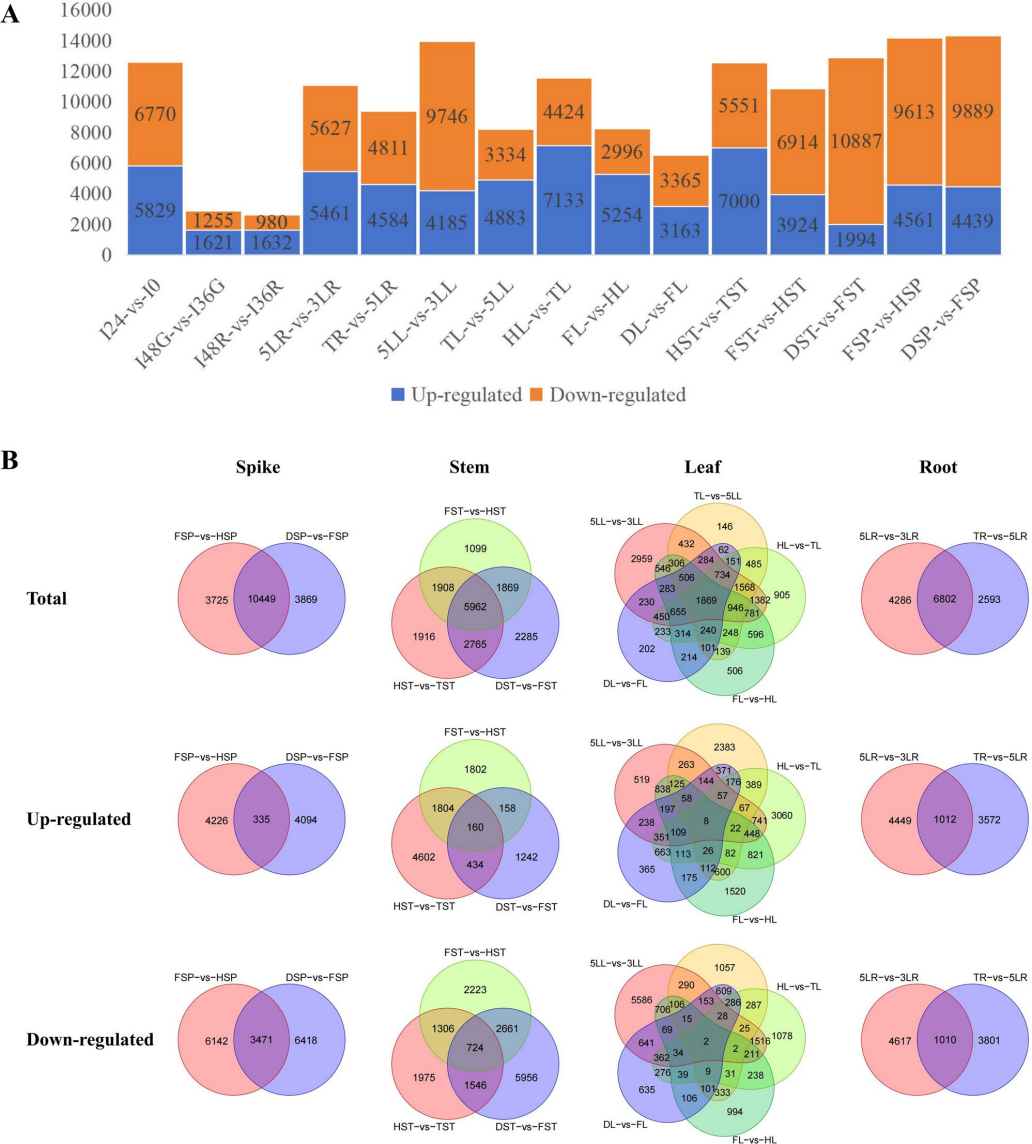
DEGs involved in the development of pearl millet. **A**, The number of up- and down-regulated DEGs. **B**, Venn diagrams demonstrate the intersection of DEGs related to the development of spike, stem, leaf, and root, respectively

The study obtained transcriptome data from multiple tissues and development stages, resulting in the identification of DEGs related to growth and development. A total of 15 groups of DEGs were analyzed, with a range of 2612 to 14,318 DEGs identified across the groups (Fig. 7A). Notably, comparisons such as I48G-vs-I36G and I48R-vs-I36R showed the least number of DEGs, while FSP-vs-HSP and DSP-vs-FSP had the highest number of DEGs (Fig. 7A). Specifically, the comparisons I48G-vs-I36G and I48R-vs-I36R exhibited the least up- and down-regulated DEGs (Fig. 7A). Conversely, groups like 5LL-vs-3LL, DST-vs-FST, FSP-vs-HSP, and DSP-vs-FSP showed the highest number of down-regulated DEGs. In terms of up-regulated DEGs, HL-vs-TL and HST-vs-TST comparisons had the highest number (Fig. 7A).

According to the continuously increased or decreased expression trends of gene in spike, stem, and root, some genes may simultaneously up- or down-regulated in adjacent sampling periods. Significant numbers of DEGs were found in comparisons like FSP-vs-HSP and DSP-vs-FSP, with an overlap of 10,449 DEGs (Fig. 7B). Furthermore, 335 and 3471 genes were simultaneously found in the up- and down-regulated DEGs in both FSP-vs-HSP and DSP-vs-FSP, respectively (Fig. 7B). Variations in gene expression were observed in comparisons such as HST-vs-TST, FST-vs-HST, and DST-vs-FST, with 5962 DEGs detected across all three groups. Co-expression analysis also revealed genes shared between up- and down-regulated DEGs in these comparisons. 160 up- and 724 down-regulated DEGs were shared by the three groups (Fig. 7B). Differential gene expression was observed in 5LR-vs-3LR and TR-vs-5LR comparisons, with 11,088 and 9395 genes showing differential expression, respectively, and 6802 DEGs being common to both groups (Fig. 7B). Additionally, 1012 and 1010 genes were simultaneously up- and down-regulated in both 5LR-vs-3LR and TR-vs-5LR, respectively (Fig. 7B).

**Fig. 8:**
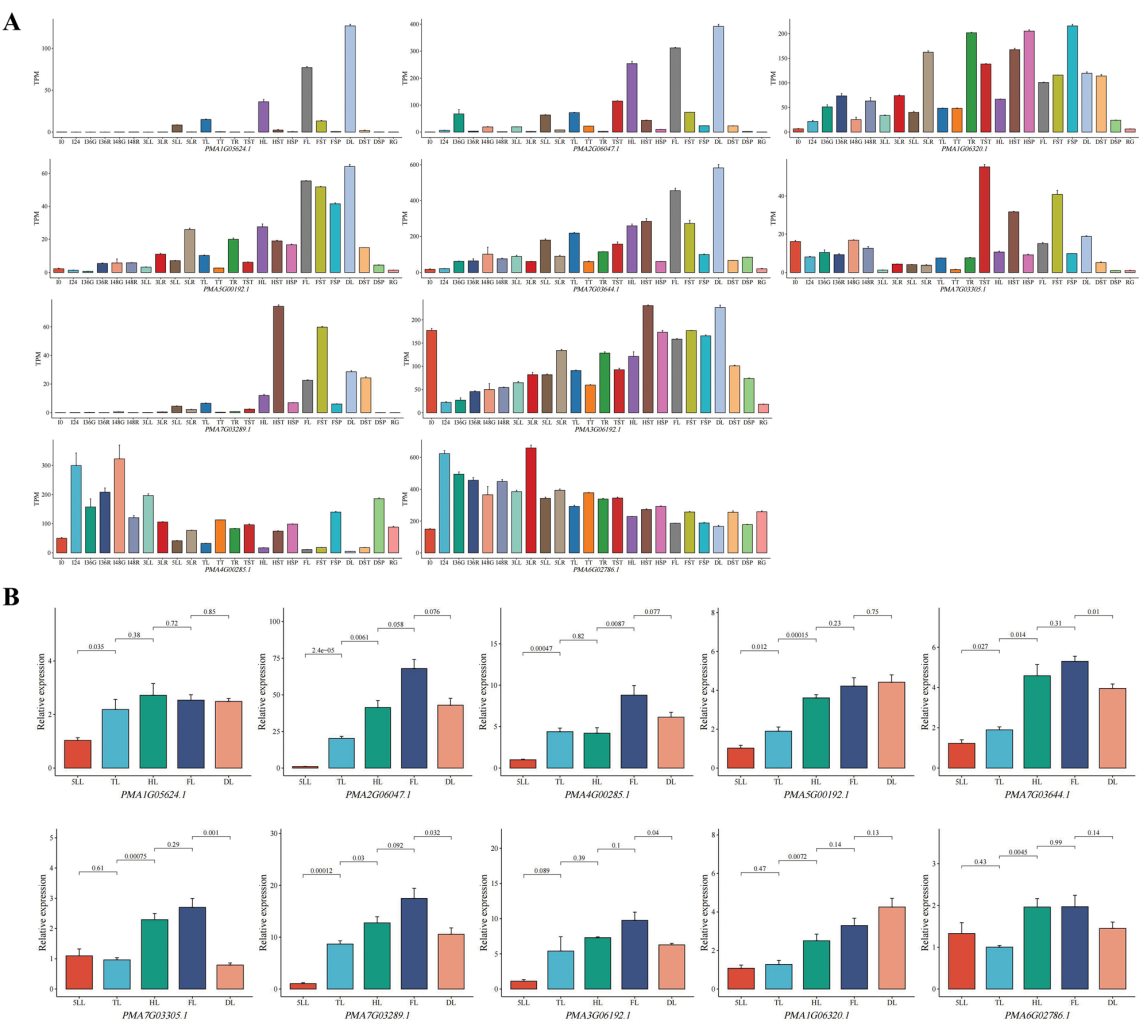
Expression patterns of 10 DEGs for different growth stages of leaf. **A**, Expression patterns of 8 continuously up-regulated and 2 continuously down-regulated genes in leaf tissues. **B**, Relative expressions of the 10 DEGs were analyzed by RT-qPCR. *PMA2G07048.1* was used as internal reference. The *p*-value was determined by the t-test

DEGs were observed between adjacent growth stages of leaf, including 13,931 (5LL-vs-3LL), 8217 (TL-vs-5LL), 11,557 (HL-vs-TL), 8250 (FL-vs-HL), and 6528 (DL-vs-FL). A total of 1869 DEGs were identified across all five groups related to leaf development, with only 8 and 2 genes being co-expressed in up- and down-regulated DEGs, respectively, across the five groups (Fig. 7B). It explained the reason why no gene expression cluster for sustained up-regulation or down-regulation were obtained in the leaf. In the context of pearl millet leaf development, the 10 genes exhibited distinct expression patterns (Fig. 8A). For instance, *PMA1G05624.1* and *PMA2G06047.1* were predominantly expressed in leaves at various growth stages, with minimal expression in other tissues. *PMA5G00373.1* showed main expression in roots and stems, while *PMA7G03289.1* exhibited high expression in stems, decreasing from the heading to maturity stage. *PMA4G00285.1* displayed varying expression levels in stems and spikes (Fig. 8A). The RT-qPCR analysis indicated that these 10 genes were expressed in leaves at various stages, with most genes upregulated before the flowering stage but downregulated at the dough stage (Fig. 8B). The expression patterns of RT-qPCR and transcriptome are not entirely consistent, which may be attributed to the different plant accessions used.

**Fig. 9:**
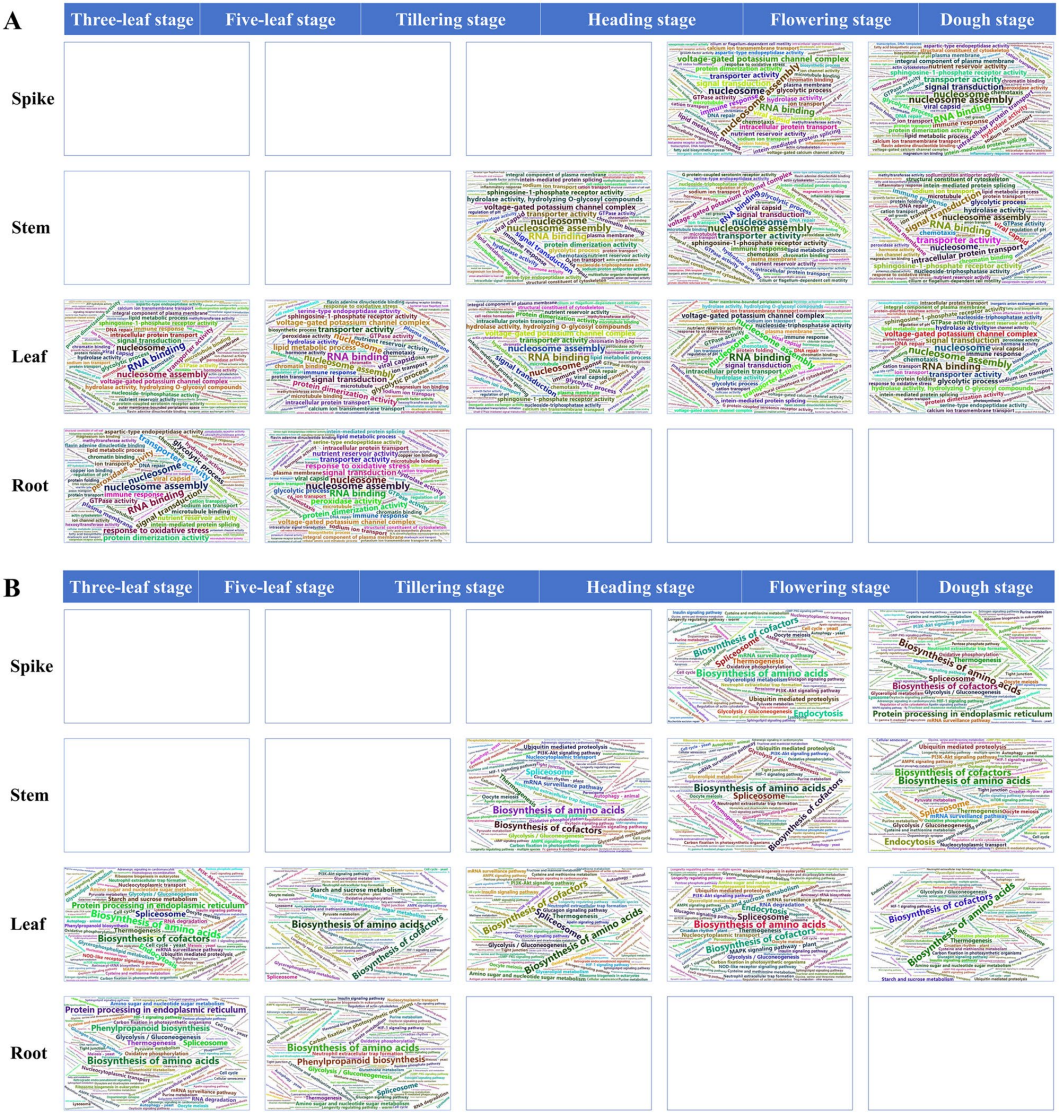
Word clouds displaying GO (**A**) and KEGG (**B**) enrichment for the DEGs involved in the development of spike, stem, leaf, and root were generated. The size of the font in the word cloud is indicative of the number of enriched genes associated with each term (pathway), with larger fonts representing a higher number of genes and smaller fonts denoting fewer genes

Enrichment results reveal that the most enriched GO or KEGG terms exhibit similarities across different tissues during the growth process. For instance, the GO term RNA binding, nucleosome assembly, nucleosome, signal transduction and transporter activity were significantly enriched among the 15 groups of DEGs (Fig. 9A). Additionally, biosynthesis of amino acids, biosynthesis of cofactors, spliceosome, protein processing in endoplasmic reticulum, endocytosis, and thermogenesis emerged as the most enriched KEGG pathways for the 15 groups of DEGs (Fig. 9B).

## Discussion

Pearl millet is a versatile crop that can be used as food, forage, and industrial plant. In this study, we conducted transcriptome analysis of 9 plant tissues across 8 growth stages to explore the HKG and TSG in pearl millet. Specifically focusing on the leaf, stem, and spike organs, which are crucial for harvesting in pearl millet, we identified DEGs involved in the growth of these organs. The findings presented in this study could serve as a valuable resource for future research and breeding efforts aimed at improving pearl millet varieties for different purposes.

### The comprehensive excavation of the HKG of pearl millet assists the study of its gene molecular function

TIRGs have played a pivotal role in gene expression normalization in previous studies; however, their reliability has lately been subjected to increased scrutiny in recent research. Czechowski et al. [19] identified *AtACT*2 and *AtTUB6* as unstable in *A. thaliana*, recommending hundreds of genes as internal reference genes for gene expression normalization across various transcript levels. In *C. quinoa*, 12 HKGs were consistently expressed across all samples, proving more stable than 2 TIRGs (*CqACT1* and *CqGAPDH*) [30]. A similar scenario was observed in pearl millet, where only 8 out of 140 members of the 6 TIRGs families (*ACT*, *CYP*, *GAPDH*, *TUB*, *UBC*, and *UBQ*) were deemed HKGs in the current study. Interestingly, *PgGAPDH* (*PMA4G00389.1*) was identified as reliable internal reference genes for accurate transcript normalization using RT-qPCR in other pearl millet accessions, and *PgUBC* (*PMA1G05188.1*) stably expressed in seedling leaves under drought or heat conditions [50–53]. These findings highlight the reliability of the HKGs identified in this research, providing more robust choices for transcript normalization in pearl millet even under stress environments.

Many studies have concentrated on analyzing housekeeping genes in specific tissues [30]. Throughout plant growth and development, genes exhibt varying expression levels at different stages. In *Arabidopsis*, only 3 genes overlapped between the top 50 stably expressed genes from seed samples by Dekkers et al. [18] and the top 100 stably expressed genes in the developmental series by Czechowski et al. [19]. It is common for a gene to have high expression levels in certain stages while showing low or no expression in others [54]. Therefore, consistently expressed housekeeping genes across various developmental stages offer more reliable options. The root, stem, leaf, and spike of pearl millet are frequently studied tissues for transcriptome and gene expression analysis, typically sampled during seedling, vegetative, and reproductive stages [8, 55–58]. This study incorporates data from these commonly studied tissues and sampling periods of pearl millet, facilitating a dynamic analysis of gene expression across multiple tissues at different developmental stages. This comprehensive analysis will significantly contribute to research on pearl millet.

The TPM values for the 7 HKGs and 4 HKG-TIRGs show some inconsistency across various TDDSs. HKGs are usually involved in essential cellular processes and are generally expected to exhibit consistent expression levels under normal conditions [28]. However, it is important to note that the stability of HKGs may differ across various tissues [59], as the concept of universally stable genes may not be applicable [60, 61]. Therefore, the relative stability of HKGs in various tissues should be considered, rather than being viewed as an absolute measure.

### Commonalities and divergences for the functions of HKGs and TSGs from different plant spices

In the 51 significant enrichment GO terms of HKGs from pearl millet, transporter activity was significantly enriched by the HKGs of maize [31], and iron-sulfur cluster binding, isomerase activity, and oxidoreductase activity, acting on the CH-CH group of donors were significantly enriched by the HKGs of *C. quinoa* [30]. In addition, 5 GO terms were significantly enriched by the HKGs of *Olea europaea* [62], including signal transduction, nucleocytoplasmic transport, protein transport, cell cycle, and vesicle-mediated transport. Furthermore, 6 KEGG pathways (spliceosome, endocytosis, oxidative phosphorylation, mRNA surveillance pathway, phagosome and proteasome) were enriched by HKGs of *G. max*, *O. europaea* and pearl millet in this study [32, 62]. These studies indicate that HKGs from different plants share some common functions, but also exhibit significant differences.

The comparative analysis of TSGs in pearl millet revealed significant differences in enrichment results of GO and KEGG pathways compared to other species. However, some TSGs were found to have similar functions across different species. For example, the TSGs for root in pearl millet, peanut and *C. quinoa* and *O. europaea* were not co-enriched with any GO term [25, 30, 62]. Three GO terms (carboxylic acid metabolic process, generation of precursor metabolites and energy, and photosynthesis, light harvesting) for leaf-specific genes were also reported in that of *C. quinoa* [30]. Two GO terms (ADP binding and hydrolase activity, hydrolyzing O-glycosyl compounds) were enriched by leaf-specific genes were identified in *C. quinoa*, *O. europaea*, and pearl millet [30, 62]. Notably, TSGs from *O. europaea* and pearl millet exhibited significant enrichment in 8 (sesquiterpenoid and triterpenoid biosynthesis, zeatin biosynthesis, MAPK signaling pathway - plant, flavonoid biosynthesis, phenylpropanoid biosynthesis, proteasome, nitrogen metabolism, and carbon fixation in photosynthetic organisms), 4 (diterpenoid biosynthesis, protein processing in endoplasmic reticulum, endocytosis, and MAPK signaling pathway), and 9 (sesquiterpenoid and triterpenoid biosynthesis, zeatin biosynthesis, carotenoid biosynthesis, flavonoid biosynthesis, pyruvate metabolism, circadian rhythm - plant, nicotinate and nicotinamide metabolism, tryptophan metabolism, and photosynthesis - antenna proteins) KEGG pathways for root, stem, and leaf, respectively [62]. Moreover, 33 KEGG pathways associated with spike-specific genes in pearl millet were enriched by flower- and fruit-specific genes in *O. europaea* [62].

*C. quinoa*, *O. europaea*, and pearl millet exhibit notable morphological and taxonomical distinctions. *C. quinoa* is an annual dicotyledonous herb from the Amaranthaceae family, *O. europaea* is an evergreen dicotyledonous tree of the Oleaceae family, and pearl millet is an annual monocotyledonous herb of the Poaceae family. The coexistence of genes with comparable functions in the same plant tissues across different plant types implies that tissue-specific genes may possess highly conserved functions. These genes could be pivotal in the development of plant tissues and represent potential targets for improving plant traits.

### Genes with stable or continuously changing expression levels during growth have the potential to improve traits

Five core genes were identified in the PPI network, playing a role in pearl millet growth from nutritional to reproductive stages. Their homologous genes exhibit significant characteristics related to reproductive growth in various plant species. For instance, *GRMZM2G002959*, the homolog of *PMA1G06601.1*, is involved in regulating leaf senescence through the jasmonic acid pathway and is associated with ear height in maize [63, 64]. Similarly, *GRMZM2G468439*, the homolog of *PMA5G04519.1*, is involved in the brassinosteroid biosynthesis II pathway, regulating the development of maize anther [65]. Additionally, *LOC_Os04g39020.1*, the homolog of *PMA3G02338.1*, is linked to aroma in rice [66]. These findings indicate that the PPI network may contribute to the development of multiple tissues in pearl millet during the reproductive growth stage.

This study investigated the transcriptomes of various tissues of pearl millet at different growth stages, leading to the identification of numerous genes that exhibit consistent up-regulation or down-regulation in roots, stems, spikes, and other tissues. These genes have the potential to contribute to trait enhancement. Pearl millet is a versatile plant used for food, forage and energy, with leaf characteristics being a crucial economic aspect. Among the eight genes that consistently up-regulate in leaves, three have homologs in other plants that serve as promising candidate genes for leaf traits or play essential roles in biological processes. For instance, the homolog of *PMA1G06320.1* in *Populus*, *AJ777792*, is a candidate gene of a QTL for leaf area [67]. Another example is *AtTHIC*, the homolog of *PMA2G06047.1*, which participates in thiamine biosynthesis in *Arabidopsis*; its mutant, *thic1*, exhibits white leaves and lethal phenotypes under normal conditions [68]. Additionally, *AtNBR1*, the homolog of *PMA3G06192.1*, encodes a selective autophagy receptor with a ubiquitin-binding domain; *Arabidopsis nbr1* mutants show increased sensitivity to various abiotic stresses such as heat, drought, and salt [69, 70]. Given that pearl millet is typically cultivated in heat and arid climates, the continuous up-regulation of *PMA3G06192.1* in pearl millet leaves from the seedling to reproductive stage could play a crucial role in enhancing the plant’s adaptation to environmental stress.

## Conclusions

In this study, we identified 461 housekeeping genes and 8091 tissue-specific genes that play crucial roles in the growth and development of pearl millet through a comprehensive transcriptome analysis of nine tissues (seed, germ, radicle, leaf, root, tillering tissue, stem, spike, and grain) across eight developmental stages in accession Tifleaf3. Eleven housekeeping genes and ten differentially expressed genes related to leaf development were validated by RT-qPCR in accession Tift23A. The findings provide valuable insights into the genetic mechanisms governing complex traits in pearl millet, offering potential targets for enhancing traits for food, forage, and energy purposes.

## Electronic supplementary material

Below is the link to the electronic supplementary material.

Supplementary Material 1: Table S1 Summary of the quality of raw sequencing data for the 24 TDDSs; Table S2 Expression (TPM) matrix of the 35486 genes in the 24 TDDSs; Table S3 Gene ID and TAU scores of HKGs; Table S4 Gene ID of the 5 gene families for TIRGs annotated in genome of PI537069; Table S5 Primers sequence; Table S6 Gene ID and TAU scores of TSGs; Table S7 Tissue-specific transcription factors.

Supplementary Material 2: Fig. S1 Expression trends of 20056 genes co-expressed in stem at tillering, heading, flowering and dough stages

Supplementary Material 3: Fig. S2 Expression trends of 19619 genes co-expressed in leaf at three-leaf, five-leaf, tillering, heading, flowering and dough stages

Supplementary Material 4: Fig. S3 Expression trends of 21373 genes co-expressed in spike at heading, flowering and dough stages

Supplementary Material 5: Fig. S4 Expression trends of 23477 genes co-expressed in root at three-leaf, five-leaf and tillering stages

## Data Availability

Expression matrix of the 35486 genes from pearl millet accession PI537069 in the multiple tissues at different growth stages was uploaded to Milletdb (http://milletdb.novogene.com/).

## Abbreviations

HKGs: housekeeping genes
TSGs: Tissue-specific genes
RT-qPCR: Real-time quantitative polymerase chain reaction
ACT: Actin
GAPDH: Glyceraldehyde-3-phosphate dehydrogenase
TUB: Tubulin
UBC: Ubiquitin-conjugating enzyme
UBQ: Polyubiquitin
CYP: Cyclophilin
TIRGs: Traditional internal reference genes
TDDSs: Tissues from differently developmental stages
CV: Coefficient of variation
TAU: Tissue specificity index
DEGs: Differentially expressed genes
TPM: Transcript per million
TFs: Transcription factors
PPI: Protein-protein interaction
I0: Imbibition - Seed
I24: Imbibition after 24 h - Seed
I36G: Imbibition after 36 h - Germ
I36R: Imbibition after 36 h - Radicle
I48G: Imbibition after 48 h - Germ
I48R: Imbibition after 48 h - Radicle
3LL: Three-leaf stage - Leaf
3LR: Three-leaf stage - Root
5LL: Five-leaf stage - Leaf
5LR: Five-leaf stage - Root
TL: Tillering stage - Leaf
TT: Tillering stage - Young tiller
TST: Tillering stage - Stem
TR: Tillering stage - Root
HSP: Heading stage - Spike
HL: Heading stage - Leaf
HST: Heading stage - Stem
FSP: Flowering stage - Spike
FL: Flowering stage - Leaf
FST: Flowering stage - Stem
DSP: Dough stage - Spike
DL: Dough stage - Leaf
DST: Dough stage - Stem
RG: Ripening stage - Grain
