# Supplementary figures and images for: Comprehensive analysis of housekeeping genes, tissue-specific genes, and dynamic regulation across developmental stages in pearl millet
Source: BMC Genomics. 2024 Dec 18;25:1199. doi: 10.1186/s12864-024-11114-3 (PMC11653590; doi:10.1186/s12864-024-11114-3)

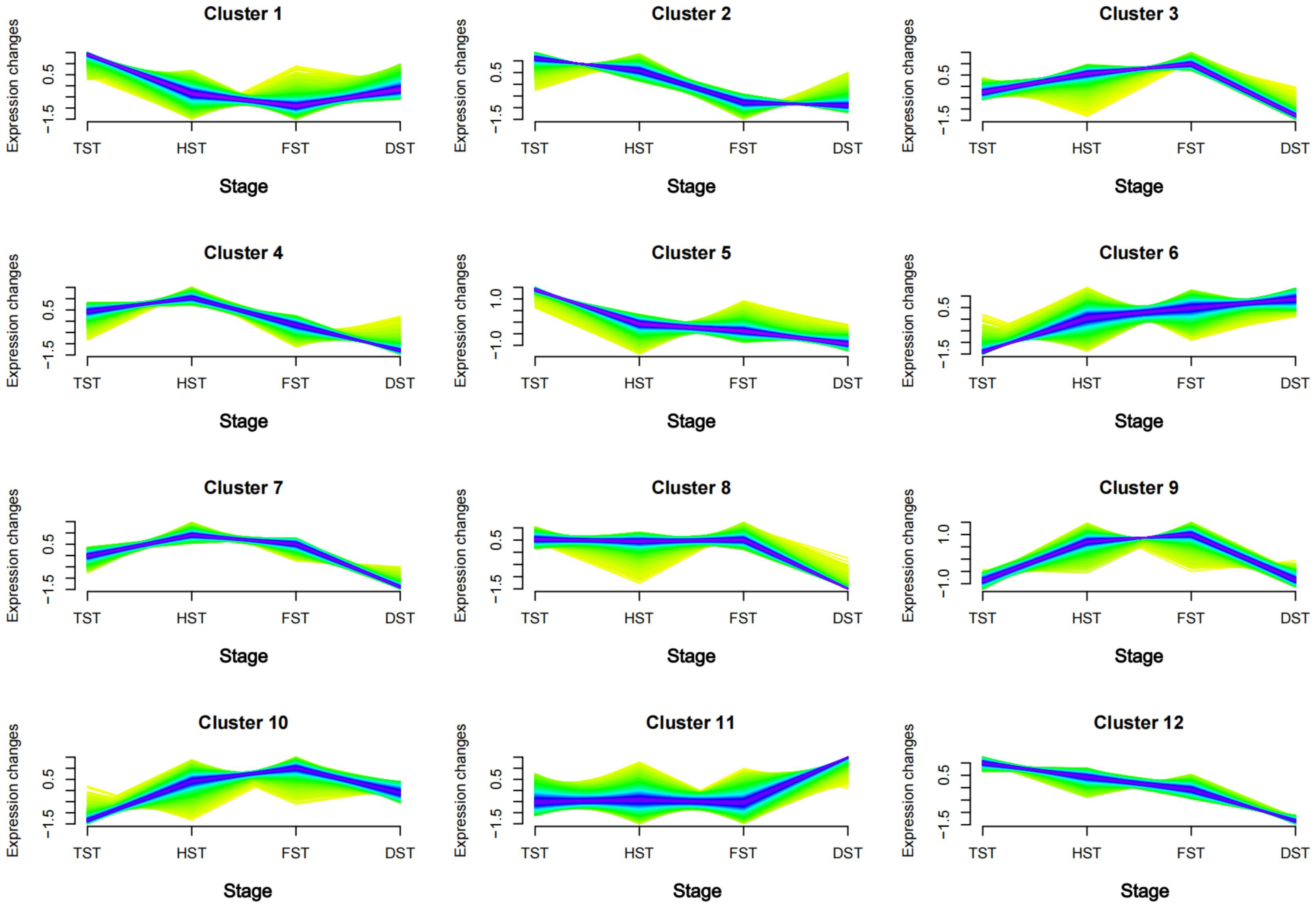

Supplement: Supplementary file 2 — Supplementary Material 2: Fig. S1 Expression trends of 20056 genes co-expressed in stem at tillering, heading, flowering and dough stages [file 12864_2024_11114_MOESM2_ESM.tif]

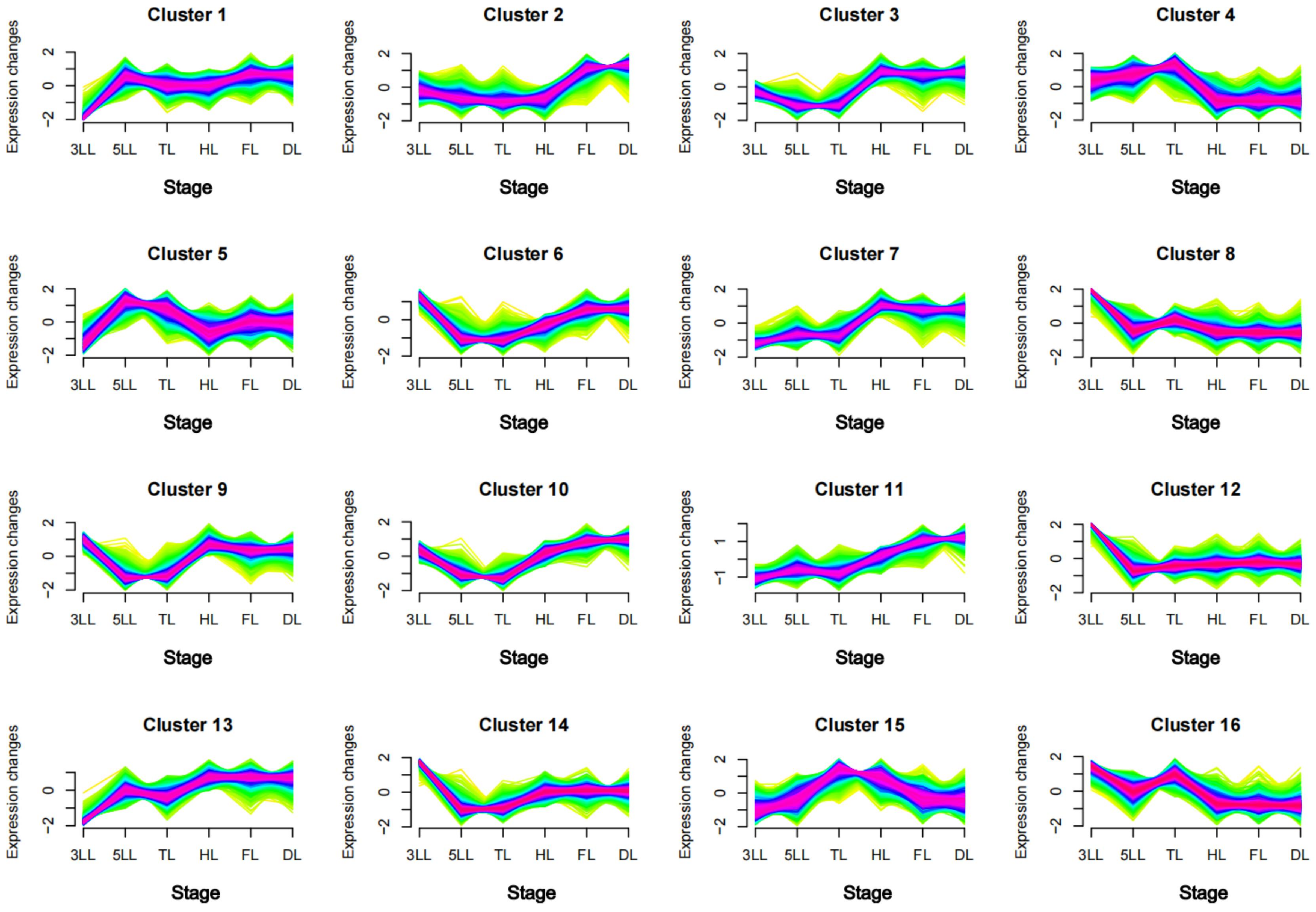

Supplement: Supplementary file 3 — Supplementary Material 3: Fig. S2 Expression trends of 19619 genes co-expressed in leaf at three-leaf, five-leaf, tillering, heading, flowering and dough stages [file 12864_2024_11114_MOESM3_ESM.tif]

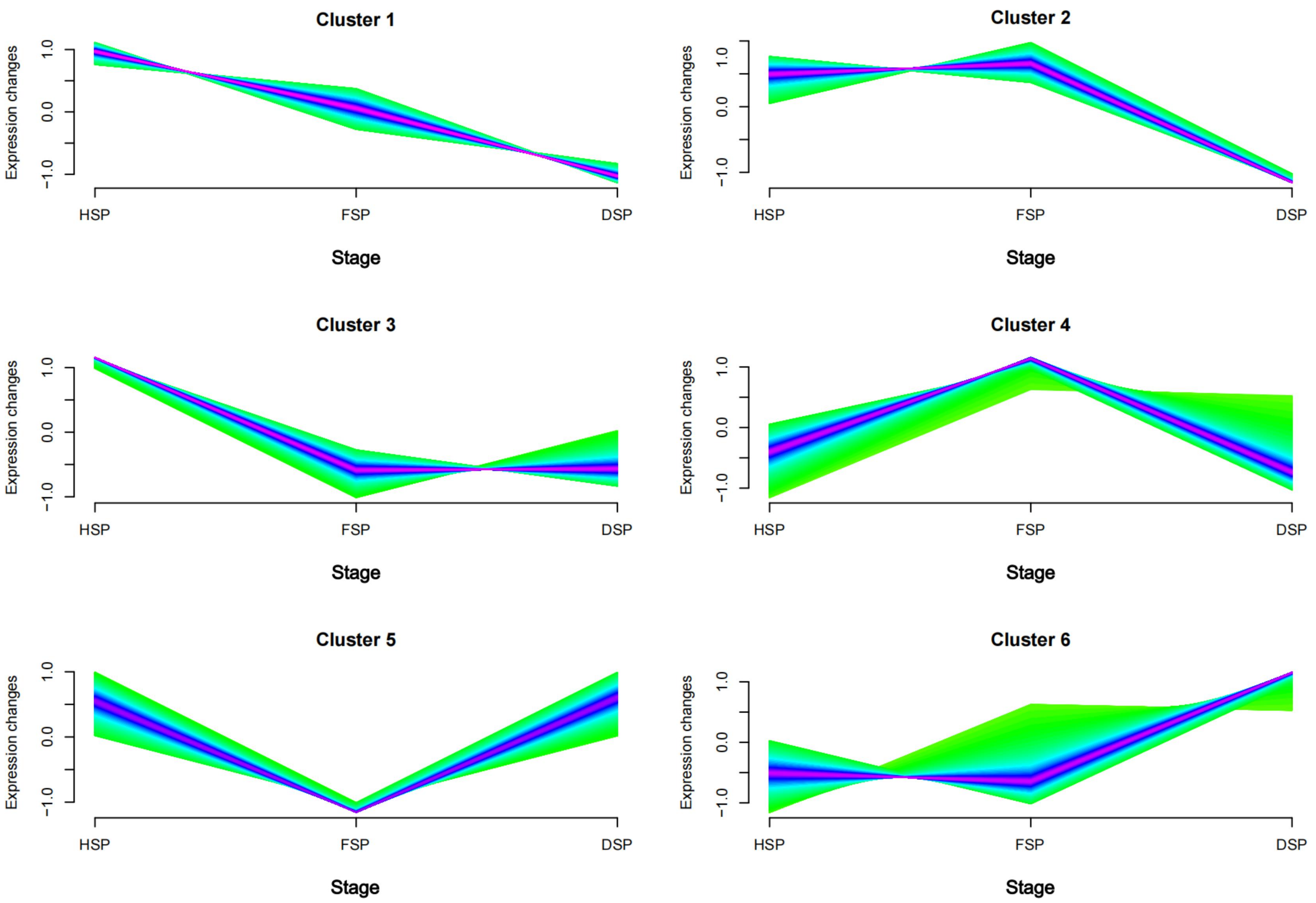

Supplement: Supplementary file 4 — Supplementary Material 4: Fig. S3 Expression trends of 21373 genes co-expressed in spike at heading, flowering and dough stages [file 12864_2024_11114_MOESM4_ESM.tif]

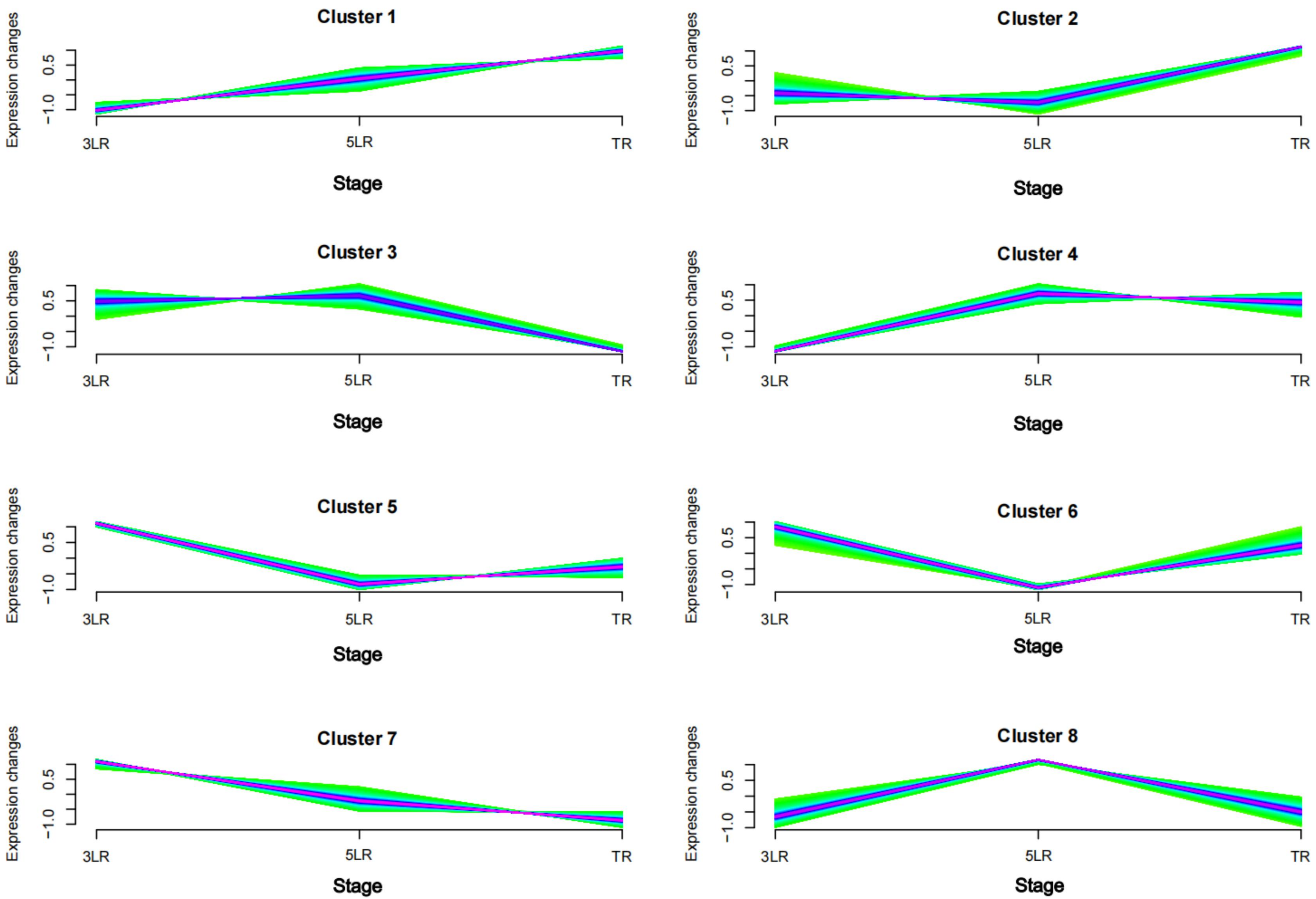

Supplement: Supplementary file 5 — Supplementary Material 5: Fig. S4 Expression trends of 23477 genes co-expressed in root at three-leaf, five-leaf and tillering stages [file 12864_2024_11114_MOESM5_ESM.tif]
